# Mitotic activity of survivin is regulated by acetylation at K129

**DOI:** 10.1080/15384101.2015.1033597

**Published:** 2015-04-30

**Authors:** Aysha M Aljaberi, Jamie RM Webster, Sally P Wheatley

**Affiliations:** School of Life Sciences; University of Nottingham; Queen's Medical Centre; Nottingham, UK

**Keywords:** apoptosis, acetylation, cancer, mitosis, survivin

## Abstract

Survivin is a cancer-associated protein regulated by multiple factors, including acetylation at K129 within its C-terminal α-helical tail. Acetylation of survivin is being pursued as a potential prognostic marker in breast cancer. This modification at K129 may cause nuclear accumulation of survivin in interphase cells; however, whether this affects its essential role during mitosis has not been addressed. We posited whether mimicking acetylation of survivin at K129 alters its activity during mitosis. Fluorescence microscopy and time-lapse imaging showed that, mutating this site to an alanine to act as a constitutive acetyl mimetic, K129A, causes defects in chromosome segregation and cytokinesis. As a non-acetylatable version, K129R, also has difficulty during mitotic exit, we conclude that cyclical acetylation and deacetylation is required for fully functional survivin during mitosis.

## Abbreviations

CPC: chromosomal passenger complex
CPP: chromosomal passenger protein
CHX: cycloheximide
DMA: dimethylenastron
IAP: inhibitor of apoptosis
NES: nuclear exportation signal
PTM: post-translational modification
SAC: spindle assembly checkpoint
SVN: survivin
WT: wild type
TRAIL: Tumor-necrosis factor Responsive Apoptosis Inducing Ligand
TSA: Trichostatin A

## Introduction

Survivin is a cancer-associated protein originally identified as a member of the inhibitor of apoptosis (IAP) family^1^ and later shown to be an essential mitotic protein.^2-4^ The pro-survival functions of survivin depend in part on its localization within immunochemically distinct pools in the cytoplasm, mitochondria, and nucleus, and on the chromosomes.^5^ Residue K129 in the C-terminal α-helical coil is acetylated by cAMP response element-binding protein, CBP, and this post-translational modification (PTM) affects its transit between cytoplasmic and nuclear compartments in interphase.^6^ However, whether this PTM impacts on its mitotic function has not been explored.

As a component of the chromosomal passenger complex (CPC), survivin regulates chromosomal movement and cytokinesis during mitosis, a role that is fundamental to genomic stability between cell generations.^2,3^ Survivin targets the CPC to the centromeres through 2 acidic residues in the BIR domain of survivin, D_70_D_71_, that bind to the NH_2_ terminus of histone H3, specifically when it is phosphorylated by the mitotic kinase, haspin.^7^ In line with its importance as the targeting subunit of the CPC, survivin is post-translationally regulated by a number of mitotic kinases, including aurora-B,^8^ cdk1,^9,10^ and plk1,^11^ and is cyclically ubiquitinated and de-ubiquitinated at several of its lysines.^12^ Failure to maintain the CPC at the centromere compromises the spindle assembly checkpoint (SAC), as monitored by BubR1, and increases the risk of genetic instability and tumorigenesis.^2,3^ The localization, function and stability of all CPPs are dependent upon the integrity of the CPC.^13^ During early mitosis, while binding to H3 by its BIR domain,^7^ the C-terminal α helix of survivin binds to the CPP, borealin.^14^ K129 is instrumental in this interaction as changing the charge at this site from the basic lysine to an acidic residue (glutamic acid, E), abrogates borealin binding *in vitro*, and expression of this form in cells causes defects in chromosome segregation.^15^ As K129E was originally identified as a single nucleotide polymorphism in a cohort of lung cancer patients,^16^ and its expression leads to genomic instability, it seemed prudent to investigate whether modification of this site by acetylation compromises survivin function. We have therefore expressed an acetyl-mimetic (K129A) version of survivin in HeLa cells and monitored the effects on cell division using fluorescence imaging, and compared them with those of cells in which global acetylation has been maintained with the deacetylase inhibitor, trichostatin A (TSA). Persistent acetylation of survivin at this site can initiate aneuploidy by compromising the SAC, allowing errors in chromosomal segregation during anaphase and failure in cytokinesis.

## Results

Two point mutations were created in the human survivin cDNA by site-directed mutagenesis, one to generate an alanine (A) substitution, and the second an arginine substitution (R) in place of lysine (K) 129 in the C-terminus of the protein. Alanine was selected as it acts as a constitutive acetyl-mimic,^6^ and arginine as a conservative mutation, but one that would be not be acetylated. All constructs were fused in frame with GFP cDNA at their C-terminus, and transfected into HeLa cells. Cell lines stably expressing these forms were selected using G418 and GFP-FACS sorting and used in all subsequent experiments.

### Localization of K129 mutants in mitotic cells

**Figure 1.**
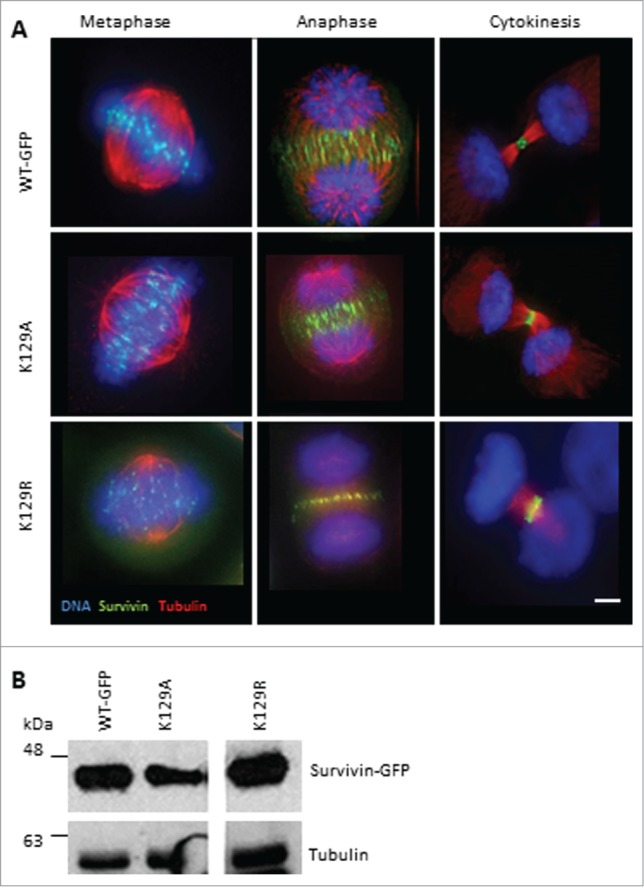
K129 mutant localization in mitotic HeLa cells. (**A**) HeLa cells stably expressing the forms of survivin-GFP (green) indicated were fixed and immunostained to reveal microtubules (red) with anti-tubulin antibodies, and counterstained with DAPI to identify the chromosomes (blue). Bar 5 μm. Black and white images of GFP signal are available in Figure S1A. (**B**) Immunoblotting of asynchronous cell lines indicated demonstrating level of expression, with tubulin used as a loading control. Note irrelevant lanes have been spliced out.

We asked whether K129 mutants localize normally during mitosis. Asynchronous cells were fixed and probed to show their microtubules (anti-tubulin antibodies) and DNA (DAPI). All versions of survivin were found at the centromeres from prometaphase through to metaphase (**Fig. 1A**). They were at the midzone microtubules between the separating chromosomes, and also at the equatorial cell cortex at anaphase. As the cells cleaved at cytokinesis, they concentrated precisely at the midbody (**Fig. 1A**, right panels). This suggests that mutating lysine 129 to alanine or arginine does not affect the mitotic localization of survivin, at least when the endogenous protein is present. Expression levels were also assessed by immunoblotting (**Fig. 1B**).

### K129A is a dominant negative mutant

**Figure 2.**
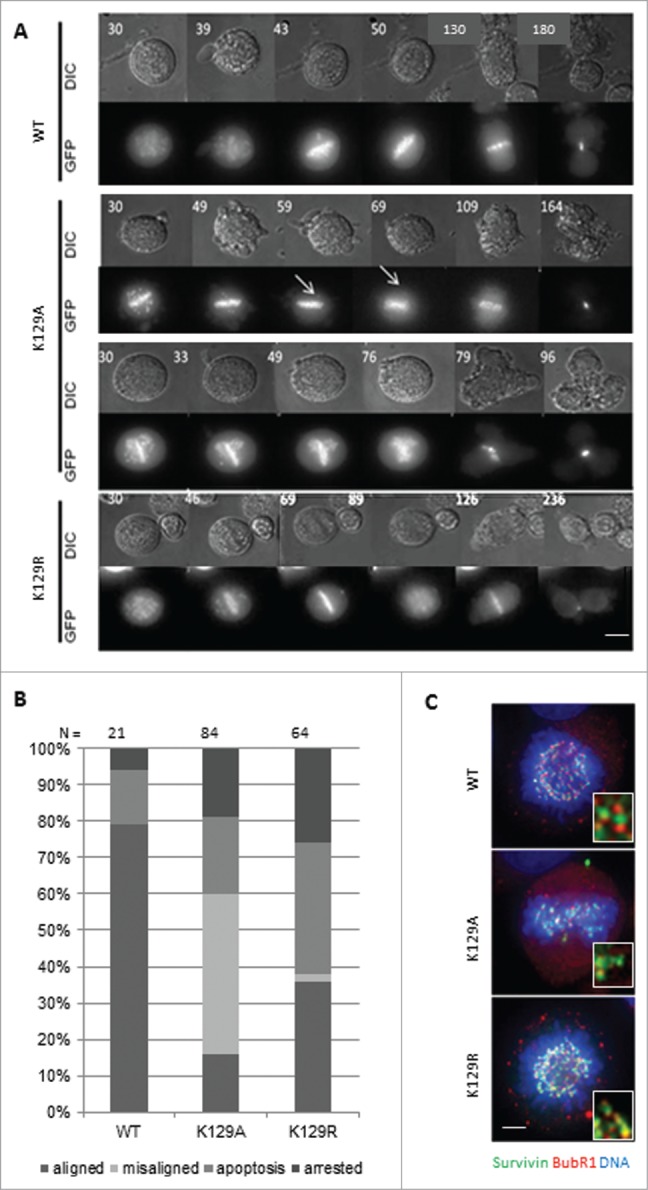
Mitotic fate of K129 mutant expressing cells. (**A**) Representative time lapse images in DIC (upper panels) and fluorescence (survivin-GFP signal, lower panels) of cells after release from DMA treatment. Time (minutes) since DMA release is indicated (top left). (**B**) Quantitation of cell fates after 12h imaging. “Aligned” – normal mitosis, “misaligned” – entered anaphase with maloriented chromosomes. (**C**) BubR1 (red) status of chromosomes in Eg5i arrested cells (6h treatment with DMA) expressing survivin variants (green), with chromosomes counterstained with DAPI (blue). Magnified regions of interest highlight BubR1 localization either side of the survivin GFP signal for WT and K129R, and its absence from some misaligned chromosomes in K129A cells. Bar 5 μm.

During mitosis, the CPC is responsible for correcting maloriented chromosomes and ensuring that SAC is satisfied before entry into anaphase. To determine whether K129 survivin mutants were functionally competent during mitosis, chromosome movements and cell fate were followed by live imaging. To exacerbate any defects that K129 mutants might have in this capacity, we treated cells with the Eg5 inhibitor, dimethylenastron (DMA), which arrests cells with monopolar spindles to which all chromosomes attach in a syntelic manner. Upon release from this reversible inhibitor, cell fate was monitored, including accuracy in anaphase and timeliness. Still images taken from the time-lapse filming are shown in **Figure 2A**, which start 30 min post-release from DMA. This experiment showed that survivin K129A could not correct all maloriented chromosomes within the cell (arrows in **Fig. 2A**). Remarkably, many cells expressing this mutant continued into anaphase, despite the persistence of misaligned chromosomes, and those that did progress and frequently failed to complete cytokinesis properly, often dividing into 3 cells instead of 2 (**Fig. 2B**). We also noted that, upon exiting mitosis (metaphase-cytokinesis), K129A cells divided more rapidly than WT cells, taking an average of 150 ± 80 min (K129A) versus 200 ± 50 min (WT). Indeed, in general, K129A grew notably faster than control cells (data not shown). In contrast, cells expressing the non-acetylatable K129R mutant took longer to reach anaphase and correct erroneously attached chromosomes, but completed division in a comparable time to WT (200 ± 35 min). While almost 80% of the divided cells of the WT went into division with corrected syntelic attachment chromosomes, >40% of the cells expressing K129A undertook division with misalignment errors and 20% underwent apoptosis (**Fig. 2B**). To assess whether the SAC was compromised cells arrested with DMA were interrogated with anti-BubR1 antibodies. While BubR1 staining was visible in all lines, importantly its absence was noted from occasional misaligned chromosomes in cells expressing K129A, suggesting that BubR1 sensing is impaired (**Fig. 2C**).

### K129A fails to support mitosis

**Figure 3.**
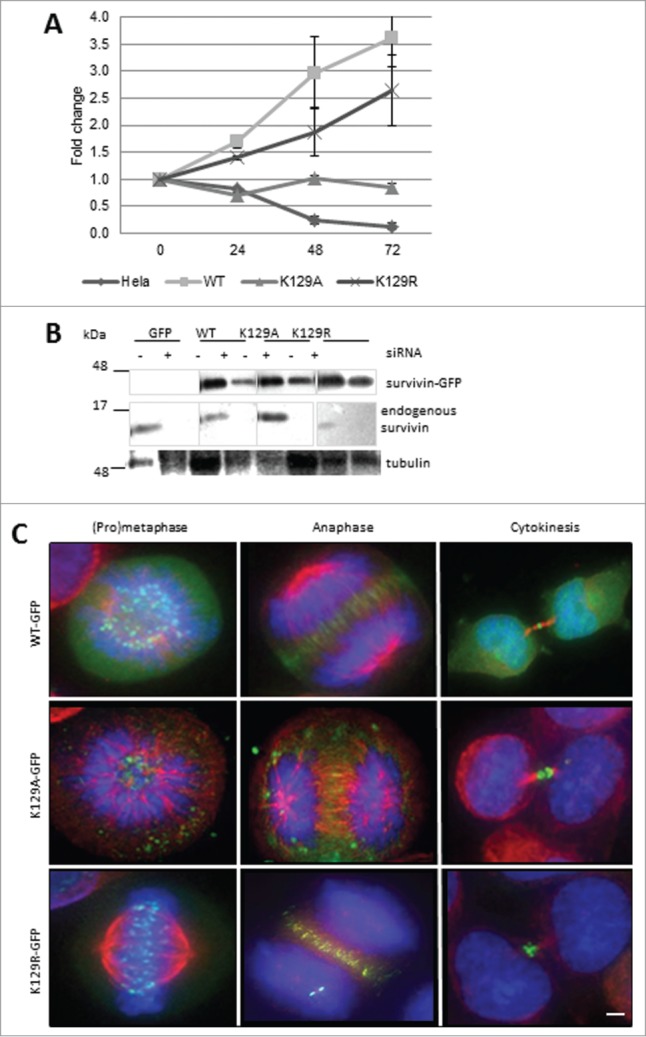
K129A cannot support mitosis. (**A**) Cell proliferation (expressed as fold increase) after siRNA removal of endogenous survivin at 24h intervals. Standard deviation of the mean is indicated for triplicates readings within a resazurin assay. Graph is representative of 3 independent repeats. (**B**) Immunoblot demonstrating efficacy of removal of endogenous survivin, and resistant expression of the ectopic forms. The blot was also probed with anti-tubulin to ensure equality of loading (data not shown). Lanes were spliced to remove irrelevant samples. (**C**) Fluorescence images of survivin variant (green) localization in mitotic cells after removal of endogenous survivin. Immunostaining reveals microtubules (red), and DAPI indicates DNA (blue). In the absence of endogenous survivin K129A localization is disrupted. Bar 5 μm. Black and white images of GFP signal are available in Figure S1B.

As survivin can bind to itself,^17^ we revisited our experiments after removal of the endogenous protein by siRNA. We asked whether these versions of survivin could support cell proliferation. In this experiment, the number of cells present in the survivin-depleted population was expressed as a percentage of the control population. After elimination of endogenous survivin from cells expressing only GFP (control), no increase in cell number occurred (**Fig. 3A**), whereas in the population expressing WT survivin, which bears a resistant mutation in the siRNA target sequence, cells continued to proliferate. Noteworthily, the population expressing K129A behaved like the GFP control, whereas those expressing K129R showed rescue more akin to WT. Efficacy of knock-down and resistance of the ectopic proteins to siRNA was confirmed by immunoblotting (**Fig. 3B**). The mitotic localization of these mutants was also re-examined. K129R localized normally, whereas K129A was no longer present at the centromeres or midzone during (pro) metaphase and anaphase, respectively (**Fig. 3C**); and when control cells were undergoing cytokinesis, K129A cells were frequently binucleated (see below).

**Figure 4.**
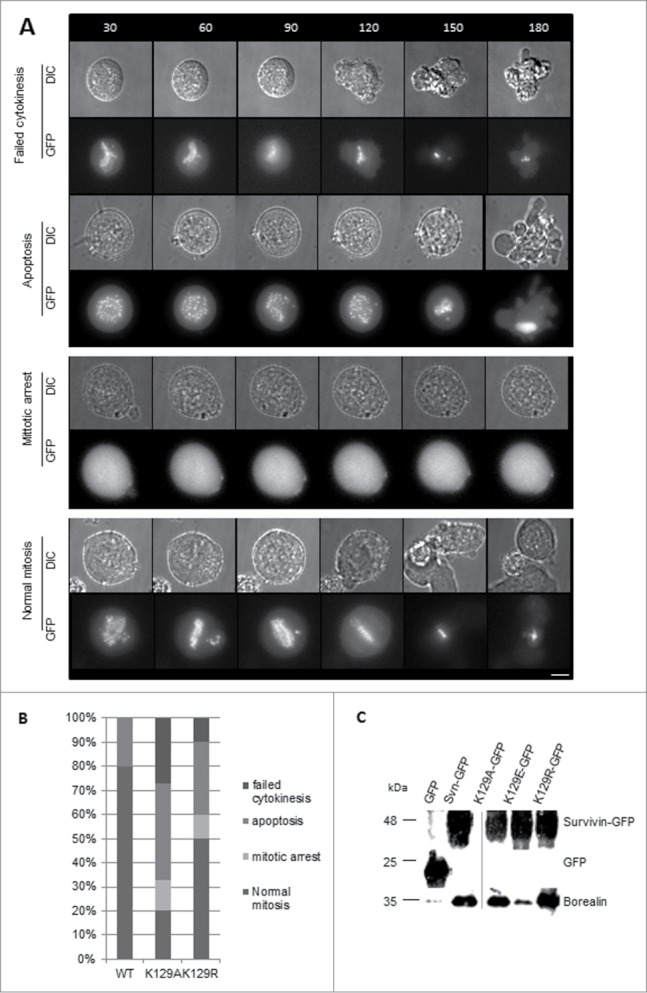
(**A**) Time lapse imaging demonstrating the fates of cells as described for **Figure 2**, but in the absence of endogenous survivin. Note that with the exception of the mitotic arrest, which shows K129A, examples are given with the K129R mutant so that the GFP signal can be observed. (**B**) Quantitation of cell fates observed in (**A**). (**C**) Co-immunoprecipitation of borealin with survivin-GFP in extracts from cell lines expressing K129 mutants. Borealin binds avidly to K129A and K129R, but K129E does not. Note, lanes 1, 2 and 4 were published in.^15^ Input for all samples is shown in **Figure S1C**.

To determine whether K129A/R expressed alone could support mitosis, the DMA arrest and release experiment was repeated 48h post-siRNA, with a number of different results. Still images of the most common fate from live imaging are illustrated using K129R cells, with the exception of the “mitotic arrest” phenotype for which a K129A cell is presented (**Fig. 4A**). Cumulative data from live imaging experiments summarised in **Figure 4B**, clearly demonstrate that K129A cannot support mitosis when expressed alone. The majority of the population either undergo apoptosis or fail to divide properly. Only 20% appear to undergo mitosis normally, and 10% arrest in prometaphase. This is in stark contrast to the 80% of WT cells dividing normally (20% do not recover from the arrest and undergo apoptosis). Although K129R expressing cells behaved relatively normally when endogenous survivin was present, only 50% of cells progressed through mitosis normally after its removal. About 10% more of this population underwent apoptosis compared with WT, and the remaining 20% either remained arrested in mitosis or failed to go through cytokinesis. Thus in the absence of endogenous survivin, K129R also causes problems, albeit less severe than K129A. These data clearly show that mitosis is impaired when either K129A or K129R is expressed.

### K129A and R mutants interact with borealin

The integrity of the CPC is critical for correct alignment of the chromosomes. Survivin is responsible for targeting the CPC to the centromeres during mitosis, which is achieved by interaction between its BIR domain and histone 3. While positioned at the centromere, survivin is also bound to borealin, which is a direct interaction involving its C-terminal α helix, and which we found to be abrogated when K129 was substituted for glutamic acid.^15^ In this regard we asked whether interaction with borealin was affected by A or R substitutions of K129. However, both K129A and K129R bound borealin avidly when co-immunoprecipitates from DMA treated cultures were examined with anti-borealin antibodies (**Fig. 4C**).

### WT survivin treated with TSA behaves like K129A

**Figure 5.**
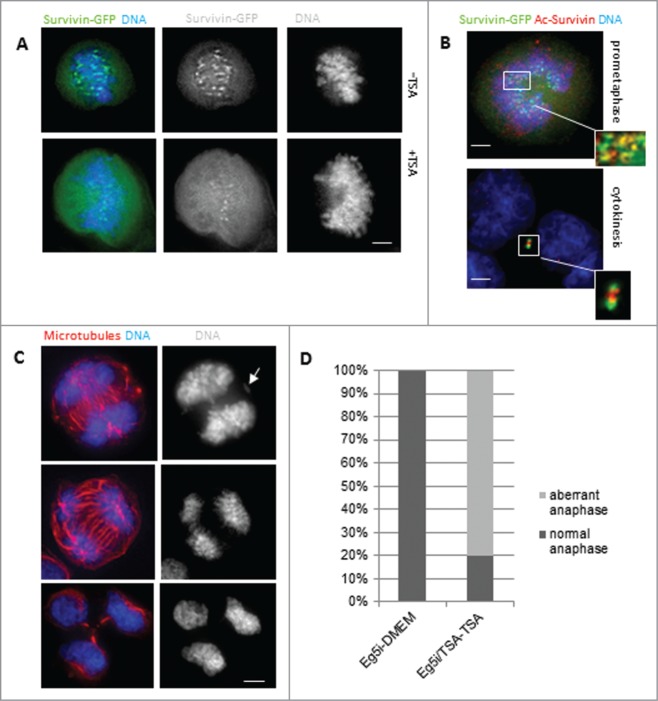
Inhibition of deacetylation with TSA. (**A**) HeLa cells expressing WT survivin were treated with TSA to prevent deacetylation, and maintain global acetylation. Survivin (green) became less focused on the centromeres and more diffusely localized when acetylation was maintained. Chromosomes were stained with DAPI (blue). (**B**) DMA induced mitotic cell expressing WT-survivin-GFP (green) and treated with TSA, then fixed and immunoprobed with anti-acetylated-survivin^K129^ specific antibodies (red), with chromosomes in blue. Acetylated survivin was less focused than total survivin (note yellow overlap as a subset of green pan-survivin staining highlighted in the inset). (**C**) Examples of aberrant anaphases (upper panels) with maloriented chromosomes (arrow), and tripolar cytokinesis (lower panel), in TSA treated HeLa cells as they exit from Eg5 inhibition. These are fixed cells immuoprobed with anti-tubulin antibodies and counterstained with DAPI. (**D**) Quantitation of cells in (C) which were treated with Eg5i or Eg5i plus TSA, and released into DMEM, or DMEM plus TSA respectively (N = 100). The majority of cells exiting mitosis in the presence of TSA made errors during anaphase. Bars 5 μm.

To link our mimetic data to acetylation *per se*, we considered whether acetylated -WT survivin behaves like K129A. Cells expressing WT survivin were treated with the deacetylase inhibitor, trichostatin A (TSA) for 6h. While WT survivin was abundant in centromeres in cells treated with DMA alone (**Fig. 5A**), it became diffusely localized throughout the cell in the presence of TSA, with a pattern similar to that of K129A (**Fig. 3C**). Immunoprobing with antibodies that specifically recognize K129-acetylated survivin indicated that the acetylated form does not localize to the centromeres, but is diffusely localized in the cell and along the chromosome arms (**Fig. 5B**, red).

When the block and release experiment was performed with DMA, in the presence and absence of TSA, approximately 80% of the TSA-treated cells had problems in anaphase (**Fig. 5C**), which accords with the mutational analysis of K129A, confirming that mitosis is impaired when survivin remains acetylated.

### Consequences of K129A expression in interphase cells

**Figure 6.**
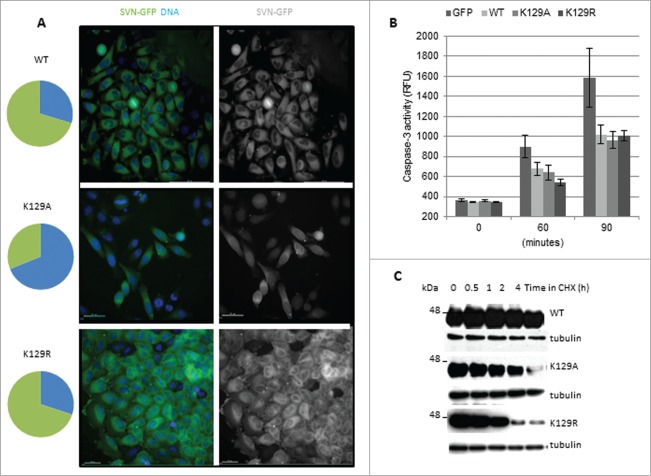
K129 mutants can inhibit apoptosis but have reduced stability. (**A**) Hela cells stably expressing WT, K129A or K129R were stained with nucleoblue and imaged live. Pie charts illustrate the percentage of cells expressing the ectopic protein in the cytoplasm (green) vs. the nucleus, or both the nucleus and the cytoplasm (blue). Bars 40 μm. (**B**) Apoptosis assay. Cells were treated with TRAIL for 0, 60 or 90 minutes, and caspase-3 activity quantitated using a tetrapeptide cleavage assay. GFP expressing cells demonstrate the level of apoptosis in control cells. All forms of survivin were able to inhibit caspase-3 effectively. (**C**) Cell lines were treated for up to 4h with CHX and whole cell lysates interrogated for expression of the ectopic proteins with anti-survivin antibodies and anti-tubulin as a loading control. Both K129 mutants had reduced stability compared to WT.

As Wang et al., (2010) reported that K129A accumulates in the nucleus, several experiments on interphase cells to corroborate and extend their findings were undertaken. We found that WT form is predominantly in the cytoplasm; K129R was also cytoplasmic, but K129A was present in both the nuclear and cytoplasmic compartments (**Fig. 6**). In many cases the signal, determined by monitoring the pixel intensity in optical slices, was higher in the nucleus of K129A cells than in the cytoplasm. Quantification (pie charts, **Fig. 6A**) refers to cells with exclusively cytoplasmic localization (green), and those in which all or some of the survivin is present in the nucleus (blue). As nuclear localization compromises the activity of survivin as an IAP,^18,19^ we tested whether K129A could still prevent cell death. Briefly, cells were treated with TRAIL for 0, 60 or 90 min, whole cell extracts prepared, and caspase-3 activity measured using a fluorogenic tetrapeptide cleavage assay. Both K129A and K129R protected cells from TRAIL as efficiently as WT (**Fig. 6B**), which establishes that K129 does not contribute to the IAP activity of survivin.

Finally, as we previously reported that nuclear survivin turns over more rapidly than cytoplasmic survivin,^19^ we investigated whether K129A compromises survivin stability. To this end cells were treated for 0–4h with cycloheximide (CHX) to inhibit protein synthesis, and lysates were interrogated for the abundance of the ectopically expressed proteins. K129A was indeed less stable than WT (**Fig. 6C**), but K129R, despite being almost exclusively cytoplasmic, also turned over more rapidly. Thus we conclude that K129 is important for survivin stability regardless of its subcellular localization.

## Discussion

Acetylation is a post-translational modification implemented by acetyl transferases/deacetylases, of which the best known are histone acetyl-transferases/deacetylases (HAT/HDACs), and a family of nicotinamide adenine dinucleotide (NAD)^+^-dependent deacetylases called Sirtuins.^20,21^ Activation of many genes is regulated by acetylation, but transcription is only one of many mechanisms regulated by reversible acetylation, and the 2 most relevant here are cell proliferation and apoptosis. For example, depletion of the ubiquitous and highly conserved HDAC3 prevents proper construction of the mitotic spindle and weakens kinetochore-microtubule attachment in prometaphase cells.^22^ Furthermore many mitotic proteins, including tubulin, BubR1,^23^ APC, shuogoshin-2,^24^ CPPs aurora-B kinase^25^ and survivin^6^ are acetylated.

Using acetylation mimetics, Wang et al., (2010) demonstrated that survivin acetylation at K129 influences its subcellular localization in interphase cells.^6^ More recently the same group has suggested that monitoring the localization of acetylated survivin has diagnostic potential in breast cancer subtyping.^26,27^ Using a similar mutagenic strategy to them, we investigated whether this PTM impacts on survivin function during mitotic exit. Our data clearly show that cells expressing the acetyl-mimetic, K129A, do not heed the “wait anaphase” signal, but rush through anaphase in the presence of maloriented chromosomes, resulting in aberrant division, often producing 3 aneuploid cells or binucleation. Thus persistent acetylation of survivin at K129 during mitosis compromises SAC and consequently cytokinesis fails, showing that if acetylated at this site, deacetylation has to occur to ensure successful segregation of the chromosomes and division.

We, like others, have demonstrated that survivin is required to ensure a robust SAC mounted by the tension sensing protein, BubR1.^2,28^ As K129A cells exit mitosis in the presence of maloriented chromosomes, this observation was extended; it showed that persistent acetylation of survivin at K129 compromises BubR1 sensing. In support of our mutagenesis data, we noted that sustained global acetylation by treatment with the HDAC inhibitor, TSA, also led to precocious entry into anaphase and cytokinesis failure. These data provide substantial evidence that the acetylation status of survivin is critical for genomic stability during mitosis. However, we are mindful that acetylation of other mitotic proteins including aurora-B^25^ and BubR1 itself,^23^ as well as certain cytokinetic proteins, may contribute to and exacerbate the mitotic and cytokinetic abnormalities seen in the chemically-induced acetylated state.

In addition to mitotic defects we found that mimicking acetylation of survivin leads to some relocation to the nucleus, which concurs with Wang et al., (2010b).^6^ By way of a mechanistic explanation, it has been proposed that survivin is acetylated in a CBP-dependent manner and that deacetylation by HDAC6^26^ aids survivin interaction with CRM1/exportin to alter its export from the nucleus.^6^ This seems unusual as survivin has a canonical NES positioned between residues 94 and 104^18,29^; however, in addition to this central NES, a second unconventional NES has been mapped from residue 119 to the end (142) of the C-terminal helix of the protein.^30^ Clearly K129 is central within this domain, and thus it seems plausible that nuclear shuttling can be regulated, at least in part, by acetylation at this site. In our experience, another consequence of nuclear expression, is accelerated clearance in a cdh1-dependent manner.^19^ Consistent with this, we found that the nuclear resident K129A was less stable than WT survivin; however, it was also noted that the predominantly cytoplasmic mutant K129R was similarly destabilised, which leads us to conclude that mutating K129 alone is sufficient to compromise its stability.

We recently reported that a charge reversal at this position, K129E, prevents survivin-borealin interaction and causes mitotic abnormalities similar to those described above.^15^ However, in marked contrast to K129E, both K129A and K129R bind avidly to borealin, eliminating the possibility that this biochemical interaction is the reason for compromising genomic integrity. Given that stability is impaired, it is possible that interaction with other non-mitotic partner, such as XIAP,^31^ are accountable for this response.

To conclude, persistent acetylation of survivin at K129 compromises the SAC, causing chromosome segregation defects and cytokinesis failure during mitotic exit.

## Materials and Methods

Tissue culture reagents were obtained from Invitrogen, tissue culture consumables from Corning, and all other reagents from Sigma-Aldrich except if noted otherwise.

### Molecular biology

Site-directed mutagenesis was used to generate point mutations using relevant primers (eurofins, MWG Operon) and Stratagene Quickchange II kit (Agilent Technologies). Wild-type human survivin cDNA bearing a siRNA resistant mutation at C54G in pBluescript was used as a template, see.^32^ Constructs were subcloned into pcDNA3.1 (Invitrogen), with a COOH-terminal GFP tag for expression in mammalian cells. Mutations were confirmed by sequencing the final constructs.

### Establishment of cell lines

HeLa cells were cultured at 37°C and 5% CO_2_ humidified incubator in Dulbecco's Modified Eagle's Medium (DMEM) supplemented with 10% fetal bovine serum (FBS), L-glutamine (2 mmol/L), 1% penicillin-streptomycin and 1% fungizome. To create cell lines stably expressing the desired constructs, HeLa cells were transfected with the pcDNA3.1 constructs using FuGENE 6 (Roche Diagnostics) diluted in Optimem, and cultured in antibiotic free medium. GFP positive colonies were selected 7–10 d post-transfection prior treatment with 500 μg/ml G418. To ensure a homogeneous population before analysis, clones were sorted by GFP positive expression FACS sorting.

### Stability assay

To test the stability of the ectopically expressed forms of survivin, cells were treated with 50 μg/ml cycloheximide (CHX) for 0, 0.5, 1, 2, or 4h, and cell lysates analyzed for survivin-GFP expression by immunoblotting with anti-GFP antibodies as described below.

### Immunoblotting and immunoprecipitation

Standard procedures were used for SDS-PAGE (12%) and immunoblotting with 0.22 μm nitrocellulose. To reveal both exogenous and endogenous survivin-GFP, nitrocellulose membranes were probed with anti-survivin antibodies (1/1000, in house) and anti-borealin antibodies (1/500, in house), and probed with anti-tubulin (B512, 1/2000, Sigma) as a loading control. Horse-radish peroxidise-conjugated from DAKO were used as secondary antibodies. Bands were detected using enhanced chemiluminescence method and X-ray film (GE Healthcare).

### Proliferation assay

Cell viability and proliferation was assessed using a resazurin assay every 24 h for 5 d. Stable cell lines expressing WT survivin and mutants were grown in 96-well plates, incubated for 1 h at 37°C with 10 μg/ml resazurin diluted in complete medium. Fluorescence emission was read in triplicate at 590 nm after excitation at 530 nm using a Fluostar Galaxy spectrophotometer.

### Immunofluorescence and fluorescence imaging

Cells were cultured on polylysine coated coverslips, fixed with 4% formaldehyde and permeabilized with 0.15% triton in PBS. Cells were blocked with 1% BSA in PBS and immunoprobed with primary antibodies for 1h at RT, before incubation with appropriate Texas-red conjugated secondary antibodies (1/200, Vector). Mitotic spindles were revealed with tubulin (B512, 1/2000, Sigma), the SAC with anti-BubR1 sheep polyclonal antibodies (1/1000, kind gift from Prof. S.S. Taylor), and borealin with an in-house antibody (1/500, rabbit). Samples were counterstained with DAPI to reveal DNA and mounted with Vectashield (Vector Laboratories). Images of fixed cells were acquired using an inverted (Olympus Axiophot, Delta Vision Elite) microscope fitted with 40x or 63x oil (NA) immersion objectives using DeltaVision software (Applied Precision). Images of 2-dimension were created from deconvolved Z-stacks (0.2 μm) and prepared using Adobe Photoshop.

### Mitotic arrest with DMA

Exponentially growing cells were treated with 2 μM dimethylenastron (DMA, Enzo Life Sciences) for 3 h before harvesting onto 35 mm, poly-L-lysine coated, glass-bottomed live image dishes (Willco) by mitotic shake-off. DMA was removed, the cells washed 3 times with warm PBS and the medium replaced with phenol-red free CO_2_ independent medium (Invitrogen)

### Acetylation experiments

To maintain proteins in an acetylated state, cells were treated for 6 h with 50 ng/ml trichostatin A (TSA). Acetylated survivin was detected in TSA treated cells using anti-acetylated^K129^ survivin antibodies (1/500, Novus Biologicals).

### Time lapse imaging

High resolution live cell imaging was carried out using Delta Vision Elite microscope fitted with 40x air objective on cells grown in 35 nm glass-boomed Willco dishes. Images of Z-sweep of 0.5 μm sections were acquired every 3 min using both GFP and differential interference contrast (DIC) filters. Subsequent analysis was carried out off-line using Volocity software (Improvision) and prepared for publication using Adobe Photoshop.

### Apoptosis assay

To assess the ability of the stably expressing mutants cells to inhibit apoptosis, caspase-3 activity was measured subsequent to inducing apoptosis with TRAIL. Cells were seeded into 24-well plates at 10^5^ per well, then incubated with 250 ng/ml with TRAIL for 0, 60, 90, 120, and 150 minutes. Cells lysed in 150 μl of mammalian protein extraction reagent (MPER, Perbio) and 1mM EDTA. Protease inhibited with pepstatin A and 4-(2-aminoethyl)-bensesulfonyl fluoride both at 1 μg/ml. 40 μl of each cell lysate was incubated in a 96-well plate with 200 μl of caspase assay buffer (20 mM HEPES at pH7.5, 10% glycerol, 1 mM Dithiothretol) and caspase-3 fluorogenic substrates Ac-DEVD-AMC (BioMol Research Labs) at 37°C for 1h. Caspase-3 activity was measured by spectrophotometry using 390 nm excitation, and 450 nm emission (Fluostar Galaxy).

### RNAi

HeLa cells stably expressing the mutants were seeded at 5 × 10^4^ cells per well of a 24-well plate immediately before RNAi transfection in antibiotic-free media. Cells were transfected with 60 pmol control or survivin specific siRNA^2^ using HyperFect (Qiagen).
